# Supplementary material for: Drug repurposing for aging research using model organisms
Source: Aging Cell. 2017 Jun 16;16(5):1006–15. doi: 10.1111/acel.12626 (PMC5595691; doi:10.1111/acel.12626)
Supplement: Supplementary file 7 — Data S1 Zip‐Archive of all report cards. [file ACEL-16-1006-s007.zip › RC_AKE.pdf]

AKE

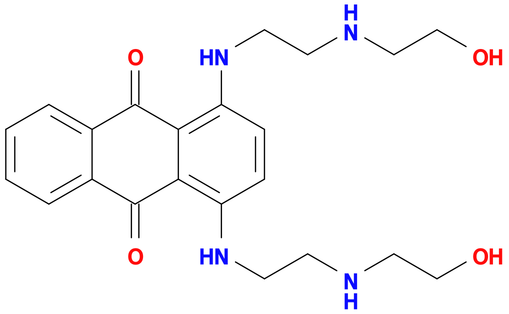

Database identifiers

ChEMBLCompound	CHEMBL49442

Ranking

	Rank	Score
Drosophila	640/697	0.122
C. elegans	567/591	0.0

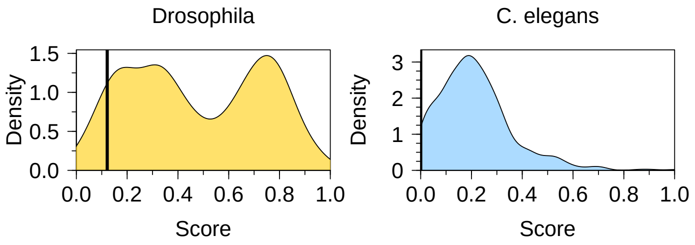

	Ageing implication	Domain conservation	Binding site conservation	Binding affinity	Bioavailability	Lipinski	Promiscuity	Purchasability	Drug approval	**Total**
Drosophila	0.624	0.963	0.843	0.377	(0.9)	-0.05	-0.0	0.0	0.0	0.122
C. elegans	0.624	0.963	0.992	0.377	0.225	-0.05	-0.0	0.0	0.0	0.0

Names

No synonyms found

Roles

ChEBI entry None has no roles

Status

Approved drug (according to ChEMBL)	No
Number of Rule of 5 violations	1
Binding affinity to original target in log units (RF-Score prediction)	4.5
Burns *C. elegans* bioavailability prediction	-3.69

Compound Target Characteristics

DNA topoisomerase 2-beta

Best gene implication in ageing for this target family came from gene F1M7A0 via mapping the annotation from RGD 62048 annotated in RGD 2014-03-11. Annotation GO 7568 (aging) was Inferred from Expression Pattern

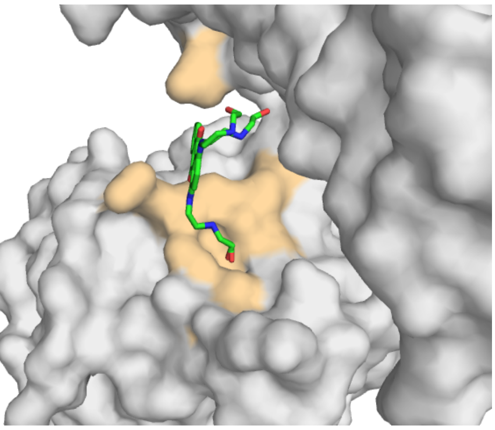

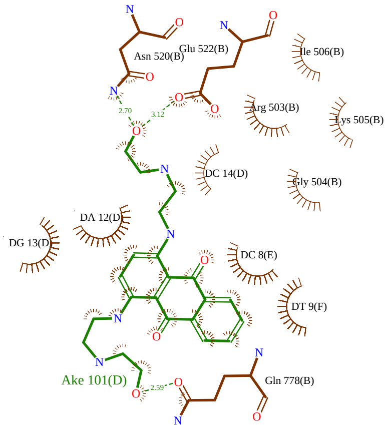

protein	amino acids contacts (binding site)
PDB:4g0w:chainA:Q02880	R G K I N E Q
tr:E9PCY5:E9PCY5_HUMAN	R G K I N E Q
sp:Q02880:TOP2B_HUMAN	R G K I N E Q
tr:F1M7A0:F1M7A0_RAT	R G K I N E M
sp:Q64511:TOP2B_MOUSE	R G K I N E Q
tr:Q6NR40:Q6NR40_DROME	R G K L N E V
sp:P15348:TOP2_DROME	R G K L N E V
sp:Q23670:TOP2_CAEEL	R G K L N E Q
tr:E9P8H0:E9P8H0_YEASX	R G K M N E Q
tr:E9P8H2:E9P8H2_YEASX	R G K M N E Q
tr:E9P8H4:E9P8H4_YEASX	R G K M N E Q
tr:E9P8H5:E9P8H5_YEASX	R G K M N E Q
tr:E9P8H7:E9P8H7_YEASX	R G K M N E Q
tr:E9P8H8:E9P8H8_YEASX	R G K M N E Q
tr:Q7LMA0:Q7LMA0_YEASX	R G K M N E Q
tr:E9P8G9:E9P8G9_YEASX	R G K M N E Q
sp:P06786:TOP2_YEAST	R G K M N E Q

protein	whole protein ident	whole protein simil	domain-based ident	domain-based simil	contact-based ident	contact-based simil
PDB:4g0w:chainA:Q02880	1.0	1.0	1.0	1.0	1.0	1.0
tr:E9PCY5:E9PCY5_HUMAN	0.71	0.71	1.0	1.0	1.0	1.0
sp:Q02880:TOP2B_HUMAN	1.0	1.0	1.0	1.0	1.0	1.0
tr:F1M7A0:F1M7A0_RAT	0.64	0.83	0.87	0.96	0.86	0.84
sp:Q64511:TOP2B_MOUSE	0.96	0.98	0.99	1.0	1.0	1.0
tr:Q6NR40:Q6NR40_DROME	0.41	0.58	0.75	0.93	0.71	0.84
sp:P15348:TOP2_DROME	0.47	0.71	0.75	0.93	0.71	0.84
sp:Q23670:TOP2_CAEEL	0.45	0.73	0.73	0.92	0.86	0.99
tr:E9P8H0:E9P8H0_YEASX	0.34	0.64	0.58	0.86	0.86	0.98
tr:E9P8H2:E9P8H2_YEASX	0.34	0.64	0.58	0.86	0.86	0.98
tr:E9P8H4:E9P8H4_YEASX	0.34	0.64	0.58	0.86	0.86	0.98
tr:E9P8H5:E9P8H5_YEASX	0.34	0.64	0.58	0.86	0.86	0.98
tr:E9P8H7:E9P8H7_YEASX	0.34	0.64	0.58	0.86	0.86	0.98
tr:E9P8H8:E9P8H8_YEASX	0.34	0.64	0.58	0.86	0.86	0.98
tr:Q7LMA0:Q7LMA0_YEASX	0.34	0.64	0.58	0.86	0.86	0.98
tr:E9P8G9:E9P8G9_YEASX	0.34	0.64	0.58	0.86	0.86	0.98
sp:P06786:TOP2_YEAST	0.34	0.64	0.58	0.86	0.86	0.98

Top2 (FBgn0003732) associated phenotypes

developmental rate defective, heat sensitive, lethal - all die before end of first instar larval stage, lethal - all die before end of second instar larval stage, lethal - all die during first instar larval stage, male fertile, neuroanatomy defective, partially lethal - majority die, small body, somatic clone

(Information from FlyBase)

Top2 (UniProt:Q6NR40) annotation

Function: Control of topological states of DNA by transient breakage and subsequent rejoining of DNA strands. Topoisomerase II makes double-strand breaks. (RuleBase:RU000381).
Subunit: Homodimer. ECO:0000256—RuleBase:RU000381.

(Information from UniProt)

Top2 (UniProt:P15348) annotation

Function: Control of topological states of DNA by transient breakage and subsequent rejoining of DNA strands. Topoisomerase II makes double-strand breaks.
Cofactor: Mg(2+) Evidence=(PROSITE-ProRule:PRU00995); Name=Mn(2+); Xref=ChEBI:CHEBI:29035; Evidence=(PROSITE-ProRule:PRU00995); Name=Ca(2+); Xref=ChEBI:CHEBI:29108; Evidence=(PROSITE-ProRule:PRU00995); Note=Binds two Mg(2+) per subunit. The magnesium ions form salt bridges with both the protein and the DNA. Can also accept other divalent metal cations, such as Mn(2+) or Ca(2+). (PROSITE-ProRule:PRU00995);
Subunit: Homodimer.
Subcellular location: Nucleus.
Miscellaneous: Eukaryotic topoisomerase I and II can relax both negative and positive supercoils, whereas prokaryotic enzymes relax only negative supercoils.

(Information from UniProt)

K12D12.1 (UniProt:Q23670) annotation

Function: Control of topological states of DNA by transient breakage and subsequent rejoining of DNA strands. Topoisomerase II makes double-strand breaks (By similarity).
Cofactor: Mg(2+) Evidence=(PROSITE-ProRule:PRU00995); Name=Mn(2+); Xref=ChEBI:CHEBI:29035; Evidence=(PROSITE-ProRule:PRU00995); Name=Ca(2+); Xref=ChEBI:CHEBI:29108; Evidence=(PROSITE-ProRule:PRU00995); Note=Binds two Mg(2+) per subunit. The magnesium ions form salt bridges with both the protein and the DNA. Can also accept other divalent metal cations, such as Mn(2+) or Ca(2+). (PROSITE-ProRule:PRU00995);
Subunit: Homodimer.
Subcellular location: Nucleus
Miscellaneous: Eukaryotic topoisomerase I and II can relax both negative and positive supercoils,

whereas prokaryotic enzymes relax only negative supercoils.

(Information from UniProt)
